# Supplementary material for: A systematic review of fuzzing based on machine learning techniques
Source: PLoS One. 2020 Aug 18;15(8):e0237749. doi: 10.1371/journal.pone.0237749 (PMC7433880; doi:10.1371/journal.pone.0237749)
Supplement: Attachment — Submitted filename: response letter.doc [file pone.0237749.s002.doc]

Dear Professors,

We owe a lot of thanks to the editors and reviewers for your reading our paper with great care and for your helpful comments. The comments are all very instructive. We have performed revisions to the manuscript according to the constructive suggestions, and we believe that the paper has been improved much. Detailed explanations are attached below. We hope it would be acceptable for publication.

Thank you again for your time and consideration.

Yours sincerely

Yan Wang, Peng Jia, Luping Liu, Cheng Huang, and Zhonglin Liu

**Answers to the Comments and Revision Explanations**

**Academic Editor:**

**Comment 1:** *Please ensure that your manuscript meets PLOS ONE's style requirements, including those for file naming.*

**Answer and Explanation 1:**

Thanks for your advice! We reviewed the format descriptions in the PLOSOne_formatting_sample_main_body.pdf and PLOSOne_formatting_sample_title_authors_affiliations.pdf documents, and referred to articles published in PLOS ONE. We have modified the format of author byline, font size, the format of headings, the format of the table, the figure citations, Finally, the name of the supporting file was modified. We used the "Track Changes" option in Microsoft Word to show these changes.

**Comment 2:** *PLOS requires an ORCID iD for the corresponding author in Editorial Manager on papers submitted after December 6th, 2016. Please ensure that you have an ORCID iD and that it is validated in Editorial Manager. To do this, go to ‘Update my Information’ (in the upper left-hand corner of the main menu), and click on the Fetch/Validate link next to the ORCID field. This will take you to the ORCID site and allow you to create a new iD or* *authenticate a pre-existing iD in Editorial Manager. Please see the following video for instructions on linking an ORCID iD to your Editorial Manager account: https://www.youtube.com/watch?v=_xcclfuvtxQ*

**Answer and Explanation 2:**

Thanks for reminding us! The corresponding author has created the ORCID iD and authenticated it on the Editorial Manager.

**Comment 3:** *Thank you for stating the following in the Acknowledgments Section of your manuscript:*

*[This work was supported by the National Key Research and Development Program of China under Grant 2017YFB0802900.]*

*We note that you have provided funding information that is not currently declared in your Funding Statement. However, funding information should not appear in the Acknowledgments section or other areas of your manuscript. We will only publish funding information present in the Funding Statement section of the online submission form.*

*Please remove any funding-related text from the manuscript and let us know how you would like to update your Funding Statement. Currently, your Funding Statement reads as follows:*

*[The author(s) received no specific funding for this work.]*

*Please include the updated Funding Statement in your cover letter. We will change the online submission form on your behalf.*

**Answer and Explanation 3:**

Thanks for reminding us! In response to your question, I have deleted the Acknowledgements part in the manuscript.

**Comment 4:** *In your Data Availability statement, you have not specified where the minimal data set underlying the results described in your manuscript can be found. PLOS defines a study's minimal data set as the underlying data used to reach the conclusions drawn in the manuscript and any additional data required to replicate the reported study findings in their entirety. All PLOS journals require that the minimal data set be made fully available. For more information about our data policy, please see http://journals.plos.org/plosone/s/data-availability.*

*Upon re-submitting your revised manuscript, please upload your study’s minimal underlying data set as either Supporting Information files or to a stable, public repository and include the relevant URLs, DOIs, or accession numbers within your revised cover letter. For a list of acceptable repositories, please see http://journals.plos.org/plosone/s/data-availability#loc-recommended-repositories. Any potentially identifying patient information must be fully anonymized.*

*Important: If there are ethical or legal restrictions to sharing your data publicly, please explain these restrictions in detail. Please see our guidelines for more information on what we consider unacceptable restrictions to publicly sharing data: http://journals.plos.org/plosone/s/data-availability#loc-unacceptable-data-access-restrictions. Note that it is not acceptable for the authors to be the sole named individuals responsible for ensuring data access.*

*We will update your Data Availability statement to reflect the information you provide in your cover letter.*

**Answer and Explanation 4:**

Thanks for reminding us! Our data mainly comes from public papers. We have uploaded the 44 papers finally selected in this manuscript to GitHub, and the URL accessed is: <https://github.com/wtwofire/A-systematic-review-of-fuzzing-based-on-machine-learning-techniques>

**Comment 5:** *Please include captions for your Supporting Information files at the end of your manuscript, and update any in-text citations to match accordingly. Please see our Supporting Information guidelines for more information: http://journals.plos.org/plosone/s/supporting-information.*

**Answer and Explanation 5:**

Thanks for your advice. we have added a caption for our Supporting Information files at the end of our manuscript, and update in-text citations to match accordingly.
